# Uretero-fallopian fistula after gynecological surgery for endometriosis: a case report

**DOI:** 10.31744/einstein_journal/2025RC1294

**Published:** 2025-06-10

**Authors:** Flavia Mansur Starling, Marcela Caetano Vilela Lauar, Gabriela Caetano Vilela Lauar, Maria Helena Pedroso, Cinthia Callegari Barbisan

**Affiliations:** 1 Beneficência Portuguesa São Paulo SP Brazil Beneficência Portuguesa, São Paulo, SP, Brazil.

**Keywords:** Urinary fistula, Fallopian tubes, Gynecologic surgical procedures, Postoperative complications, Wounds and injuries, Laparoscopy, Video recording, Magnetic resonance imaging, Tomography, X-ray computed

## Abstract

The increasing use of videolaparoscopy for the complete excision of endometriotic lesions and restoration of pelvic anatomy has helped improve long-term outcomes. However, risks remain, including rare but significant complications such as salpingoureteral fistulas. Early diagnosis using imaging methods enables adequate treatment and reduces the risk of new complications. In this report, we present a case of uretero-fallopian fistula following gynecological surgery for the resection of endometriotic lesions, with intraoperative inadvertent ureteral injury. This case report aimed to highlight the importance of diagnostic imaging in postoperative complications of gynecological surgeries for endometriosis, especially if inadvertent injuries occur during the procedure, and to guide the medical community in suspecting and addressing this condition.

## INTRODUCTION

Videolaparoscopy with complete excision of lesions and restoration of the pelvic anatomy has been increasingly adopted for the treatment of endometriosis, leading to improved long-term outcomes. However, such surgeries carry the risk of complications, including urinary issues. Although rare, some significant complications such as salpingoureteral fistula can arise, requiring careful management.^(1)^

Urogenital fistulas are abnormal communications between the female genital tract and the bladder, urethra, or ureters.^(2)^ As the name suggests, a uretero-fallopian fistula is a fistula between the ureter and the fallopian tube. It is a rare complication, with approximately seven cases reported in the literature to date, most often occurring as a consequence of gynecological or urological surgeries.

Most ureteric injuries are iatrogenic, with up to 73% of cases being gynecological.^(3)^ Early recognition is highly important for the management and favorable outcome of postoperative ureteral injuries, and diagnosis usually requires radiological studies, such as cross-sectional modalities.^(4)^ However, these injuries may not be immediately evident and may only become noticeable days after the surgery, once the patient has exhibited signs and symptoms.^(5)^

## CASE REPORT

**Figure 1:**
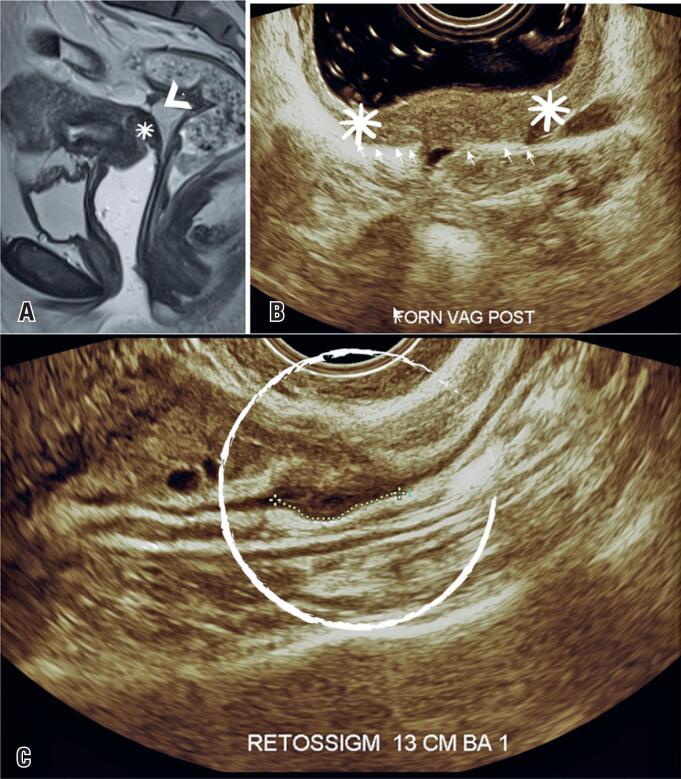
(A) Magnetic resonance imaging of the pelvis in sagittal T2WI (T2-weighted image), with no fat saturation and showing thickening with low signal on the retrocervical region (white head arrow) and vaginal fornix (asterisk), which is related to endometriosis lesions. (B and C) Transvaginal ultrasound with bowel preparation showing invasion of the vaginal mucosa (asterisks) and rectosigmoid deep infiltrating endometriosis (white circle)

**Figure 2:**
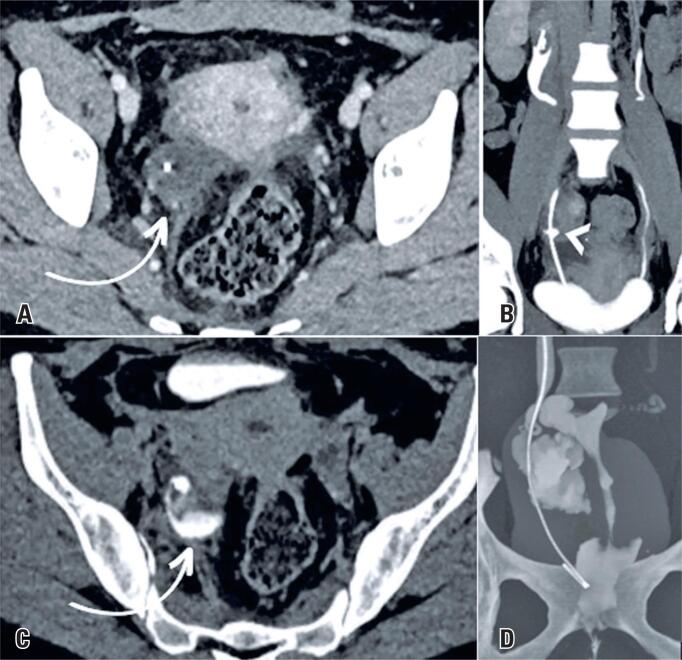
Axial (A and C) and coronal (B) contrast-enhanced CT scans showing a hypodense collection on the right of the pelvic excavation (white arrows), with a component in the adnexal region and near the lower third of the ureter, identifying extravasation of contrast in the excretory phase in the lower third of the ureter (arrowhead) that fills the mentioned collection, suggesting urinoma (C). Coronal MIP contrast-enhanced CT scan (D) showing extravasation of contrast into the collection and into the right uterine tube, suggesting a uretero-fallopian fistula, with consequent passage of contrast into the uterine cavity and vagina

A 27-year-old female patient underwent videolaparoscopy in 2022 for resection of endometriosis involving the retrocervical region, uterosacral ligaments, posterior vaginal fornix, rectosigmoid, and origin of the left round ligament, which was diagnosed through imaging tests, including magnetic resonance and transvaginal ultrasound with bowel preparation (Figure 1A-C). During the procedure, an inadvertent injury to the right ureter occurred. The urology team was requested and performed a segmental ureterectomy with uretero-ureteral anastomosis and placed a double J-stent. Two days after hospital discharge, the patient sought emergency care, complaining of right abdominal and lumbar pain and dysuria. Computed tomography (CT) revealed a hypodense collection adjacent to the distal third of the right ureter filled with excreted iodinated contrast in the late phase, which was compatible with a fistula (Figure 2A-C). The patient was admitted to the hospital, and an indwelling bladder catheter was inserted along with antibiotic therapy administration. After 10 days, a new CT scan was performed, which showed dimensional stability of the liquid collection in the right pelvic excavation. The scan continued to show extravasation of contrast into the collection with serpiginous accumulation of contrast in the right adnexal region, suggesting a uretero-fallopian fistula. This led to the passage of contrast into the uterine cavity and vagina and a small accumulation of contrast in the left fallopian tube (Figure 2D). The patient then underwent a new robotic surgical procedure, which involved right ureteral reimplantation with a Boari flap, psoas hitch, and placement of a double J-stent.

Two years after reconstructive surgery, the patient was contacted and reported to be satisfied with the resolution of the complications. She emphasized receiving excellent care from the entire medical team. However, the loss of health insurance prevented her from scheduling follow-up examinations or consultations. Despite this, the patient stated that she was in good health, with no further issues related to endometriosis or surgery and experienced almost complete improvement of her symptoms.

## DISCUSSION

Ureteral injuries are most commonly iatrogenic in origin, and uretero-fallopian fistulas are rare complications that are usually secondary to gynecological or urological surgeries.^(4,5)^

During gynecological surgery, the ureter is at risk of injury not only because of its close proximity to pelvic structures, such as the rectosigmoid and uterocervical junction, but also because of the biological variability in its location. Ureteral injuries can occur via contusion, devascularisation, kinking, laceration, clip application, suture ligation, or transection. Factors that increase the risk of ureteral injury include an abnormal pelvic anatomy, presence of endometriosis, development of pelvic adhesions and/or large adnexal masses, and unexpected occurrence of intraoperative bleeding.^(6)^

While many cases of fistulas between the fallopian tubes and bowel have been reported in the literature, very few involve the fallopian tubes and the urinary tract. This condition can occur after major surgeries, such as resection of rectal cancer^(7)^ or open ureterolithotomy^(8)^ as well as videolaparoscopy for endometriosis resection.^(5,7)^

The intraoperative diagnosis of ureteral injuries is challenging and is delayed in 90% of cases. Therefore, careful attention is crucial when a patient has severe abdominal pain, sepsis, and/or renal insufficiency or if urine is present in drains.^(9)^

Women affected by a ureteral fistula in the female reproductive tract, such as the fallopian tube, may experience pelvic pain and vaginal urine discharge, and a pelvic examination can reveal urine leakage from the cervical os.^(10)^ Diagnosis can be established radiographically and intravenous contrast-enhanced CT should be performed promptly upon clinical suspicion to aid in the diagnosis,^(11)^ for preoperative evaluation and surgical planning.^(10)^

## CONCLUSION

Intraoperative diagnosis of fistulas in the female reproductive tract is challenging, and imaging plays a crucial role in their detection. By defining the anatomy of the fistula and its extent, cross-sectional modalities such as CT, magnetic resonance imaging, and ultrasonography have attracted increasing attention not only for diagnosis but also for treatment planning. Therefore, radiologists should be familiar with the radiological features of genitourinary tract fistulas to ensure accurate diagnosis and treatment planning.
